# Oxidative Stress Effects of Soluble Sulfide on Human Hepatocyte Cell Line LO2

**DOI:** 10.3390/ijerph16091662

**Published:** 2019-05-13

**Authors:** Ying Shao, Zhongli Chen, Lingling Wu

**Affiliations:** 1 Institute for Environmental Research (Bio V), RWTH Aachen University, Worringerweg 1, 52074 Aachen, Germany; ying.shao0926@outlook.com; 2 Key Laboratory of the Three Gorges Reservoir Eco-environment, Ministry of Education, Chongqing University, Chongqing 400045, China; 3 Key Laboratory of Yangtze River Water Environment, Ministry of Education, Tongji University, Shanghai 200092, China

**Keywords:** soluble sulfide, oxidative stress, cytotoxicity, superoxide dismutase (SOD) activity, glutathione peroxidase (GSH-Px) activity

## Abstract

Soluble sulfide is well known for its toxicity and corrosion for hundreds of years. However, recent studies have demonstrated that hydrogen sulfide (H_2_S)—a novel gasotransmitter—supports a critical role during neuromodulation, cell proliferation, and cardioprotection for organisms. In particular, soluble sulfide plays multifaceted signaling functions in mammals during oxidative stress processes. However, the specific molecular regulation of soluble sulfide during oxidative stress remains unclear. In this study, Na_2_S was implemented as a soluble sulfide donor to expose LO2 cells. The 3-(4,5-dimethylthiazolyl-2),-2,5-diphenyltetrazolium bromide (MTT) assay, hydroxyl radical assay, superoxide dismutase (SOD) assay, and glutathione peroxidase (GSH-PX) assay were applied to analyze cytotoxicity, hydroxyl radical levels, SOD and GSH-Px activities, respectively. Soluble sulfide at a concentration 0.01–1.0 mM/L resulted in a marked and concentration-dependent reduction of LO2 cell viability. At low concentrations, sulfide solutions increased SOD activity and GSH-Px activity of LO2 after 24 h exposure, exhibiting a clear hormesis-effect and indicating the protective ability of soluble sulfide against oxidative stress. The decline in SOD and GSH-Px and the increase in hydroxyl radical (0.08–1.0 mM/L) suggested that oxidative damage could be a possible mechanism for sulfide-induced cytotoxicity.

## 1. Introduction

Sulfide, as an inorganic anion of sulfur, is found in concentrations ranging from μg/L to mg/L in the surface water [1], sewers [2], waste water, waste water treatment plants [3], and biological fluid such as rat blood [4]. In the aquatic environment, soluble sulfide which has been known for its toxicity and corrosion for hundreds of years normally coexists as sulfide ion (S^2−^), hydrosulfide (HS^−^) and hydrogen sulfide (H_2_S) [3]. In particular H_2_S, one of the most toxic chemicals in the end of Permian period, is recognized as the root cause for multiple mass extinctions on earth [5]. Moreover, dissolved free sulfides by means of H_2_S, HS^−^, and S^2−^ cause strong aggressive corrosion of many metals, which is a major process affecting sewer systems, leading to very high maintenance costs worldwide [6].

Recent studies have illustrated that H_2_S has emerged as a novel gaseous signaling molecule (gasotransmitter), which is as important as nitric oxide (NO), carbon monoxide (CO), and hydrogen peroxide (H_2_O_2_) [7,8] for animals and plants. H_2_S, as a physiologic mediator, is found to participate in many physiological and pathological processes in various organisms. It has been identified in cardiovascular, immune, and nervous systems during apoptosis [9]; during inflammatory processes, protective effects against hypoxia, neuromodulation, cell proliferation and cardioprotection [8,10]. Therefore, public concerns were received regarding H_2_S worldwide [11]. In 2011, an European network on gasotransmitters (ENOG) was formed to promote research on H_2_S, aiming to unravel its roles in human health and disease [12], and achieved many results. For instance, H_2_S is involved in smooth muscle relaxation that causes penile erection, thus presenting possible new therapeutic opportunities for erectile dysfunction [13]. H_2_S, as a smooth muscle relaxant, can relax blood vessels via activating ATP-sensitive potassium channels in smooth muscle cells [14]. Exogenous H_2_S induces the decline in blood pressure and the progression of nephropathy in mammals [15]. H_2_S serves as a component of vasodilators that is potentially protecting against cardiovascular disease [11]. H_2_S stimulates the increased response of N-methyl-D-aspartate receptors and facilitates long-term potentiation, which may influence memory formation [16]. NaHS at environmental concentrations of 30–50 µmol/L could reduce the homocysteine-induced cytotoxicity in vascular smooth muscle cells [17]. It is noticeable, however, that soluble sulfide, as a reducing substance, plays an important role during oxidative stress regulation within organisms. A cystathionine gamma-lyase study reported that H₂S reduction may contribute to cisplatin-induced renal cell injury, which is possibly caused by the augmentation of endogenous reactive oxygen species (ROS) production. NaHS pretreated PCl_2_ cells have been found to inhibit ROS production through cobalt chloride (CoCl_2_) [18]. Hydrogen sulfide protects the integrity of the blood–brain barrier following cerebral ischemia possibly by inhibiting free radical production [19]. Even though the protective effects of low dose H_2_S on neurons and cardiac muscles against oxidative stress have been frequently observed in many studies [20,21], a data gap remains with regard to the specific regulation mechanism of soluble sulfide during oxidative stress in human hepatocyte cells. Thus, the research questions guiding this study were: 1) how can soluble sulfide regulate oxidative stress in human hepatocytes? 2) Does soluble sulfide have any antioxidant capacity? If yes, 3) at what concentration does soluble sulfide induce antioxidantion? Considering the toxicity of soluble sulfide, is there any linkage between oxidative stress and cytotoxicity at high doses of soluble sulfide exposure?

To answer the above questions, we used the human hepatocyte cell line LO2 was used to test the cytotoxicity and the regulatory effects of soluble sulfide. The LO2 cells have been reported to be an ideal alternative to in vivo tests with intact hepatocyte enzyme activities, which closely resemble the enzyme activity levels of the current population [22]. Na_2_S, a soluble sulfide donor [3,23], was used to expose LO2 cells to different concentrations. We also used the 3-(4,5-dimethylthiazolyl-2),-2,5-diphenyltetrazolium bromide (MTT) assay, an important in vitro method for toxicological analysis [24], to assess the cytotoxic potential of soluble sulfide. This assay determines cell viability in terms of reductive activity by measuring the enzymatic conversion of tetrazolium compounds to water-insoluble formazan crystals by dehydrogenases found in the mitochondria of living cells [25]. Oxidative stress reflects the imbalance between the systemic manifestation of reactive oxygen species (ROS) including peroxides, superoxide, hydroxyl radical, singlet oxygen, and alpha-oxygen [26]. Toxic effects could be induced by disturbing the cells’ normal redox state through the production of peroxides and free radicals, leading to damage in the whole cell components, including proteins, lipids, and DNA [27]. To analyze the specific regulatory effects of soluble sulfide on oxidative stress, ROS including hydroxyl radical, superoxide and peroxides were detected by the hydroxyl radical assay, superoxide dismutase (SOD) assay, and the glutathione peroxidase (GSH-PX) assay after 24 h of exposure, respectively [28,29]. These oxidative stress tests and cytotoxicity assay were performed at different sulfide concentrations to explore the specific mechanism of sulfide induced oxidative damage. 

## 2. Materials and Methods

### 2.1. Materials

Na_2_S·9H_2_O (98%) was purchased from Shanghai TongYa Chemical Industry Science and Technology Co. Ltd. The NaOH and HCl used to adjust pH values were obtained from Sinopharm Chemical Reagents Co. Ltd (Shanghai, PR China). In this study, we used double-distilled water passed through a reverse osmosis system and further treated with a Hitech-K flow water purification system.

Dulbecco’s modified Eagle’s medium (DMEM), fetal bovine serum (FBS), and 0.25% trypsin were purchased from KeyGen BioTECH. Co. Ltd. 3-(4,5-Dimethylthiazol-2-yl)-2,5-diphenyl tetrazolium bromide (MTT) was provided by the Nanjing Jiancheng Bioengineering Institute. Antibiotic–antimycotic and DMSO were produced by the Institute of Nanjing Jiancheng Biology Engineering (Nanjing, PR China). Bovine serum was provided by Hangzhou Sijiqing Biological Engineering Co. Ltd. (Hangzhou, PR China). All other chemicals were purchased from Nanjing Ronghua Reagent Co. (Nanjing, PR China).

### 2.2. Cell Culture

The human normal hepatocyte LO2 cells were purchased from KeyGen BioTECH. Co. Ltd. (Nanjing, PR China). Cell culture was conducted according to the method described by Tang et al. [30], with slight modifications. The cells were grown in Dulbecco’s modified Eagle’s medium (DMEM) supplemented with 10% (v/v) fetal bovine serum (FBS) and 1% antibiotic-antimycotic, in polystyrene flasks (75 cm^2^, Corning Incorporated) at 37 °C in 5% CO_2_. The media was changed every 2 days. LO2 cells were maintained in growth phase by splitting every 3 days using 0.25% (v/v) trypsin-ethylene diamine tetraacetic acid (EDTA, 1 mM).

### 2.3. The MTT Assay

Cytotoxicity of soluble sulfide in LO2 cells was tested using the MTT assay. The MTT assay was conducted according to the protocol described by Storch et al. [31], with slight modifications. LO2 cells were cultured in DMEM (Gibco, Thermo Fisher, Germany) in 96-well plates at 10,000 cells/well density, and were incubated for 24 h at 37 °C in 5% CO_2_. The cells were then exposed to soluble sulfide solutions at concentrations of 0.01, 0.05, 0.1, 0.2, 0.5, and 1.0 mM/L at 37 °C for 24 h. After incubation, 100 μL of MTT (0.5 g/L with reagent in D-MEM medium) was added to each well and the cells were incubated for 30 min at 37 °C. The MTT solution was then removed, 200 μL of DMSO was added, and the plates were incubated for 15 min at 37 °C to dissolve the formazan crystals. Finally, the absorbance of each well was immediately measured on an ELISA micro-plate reader (BIO-RAD, USA) at 550 nm to determine cellular viability.

### 2.4. Measurement of the Hydroxyl Radical

Hydroxyl radicals were generated by a Fenton reaction (Fe^3+^-ascorbate-EDTA-H_2_O_2_ system), and the scavenging capacity towards hydroxyl radicals was measured using a deoxyribose method previously described by Szabo et al. [32] with slight modifications. Reaction mixtures consisted of 1.0 mM/L deoxyribose, 1.0 mM/L H_2_O_2_, 1.0 mM ascorbate, and 1.0 mg/ml of particles in a total volume of 2 ml. In control samples, the mixtures were replaced by PBS. The mixtures were incubated at 37 °C for 1.5 h with agitation and then centrifuged at 1200× g for 10 min. TBA (1 ml; 1%, w/v) and trichloroacetic acid (2.8%, w/v) were added to each of the supernatants (1 ml), followed by heating at 37 °C for 3 min [33]. The reaction produced a pink chromogen and was quantified by measuring absorbance at 530 nm. 

### 2.5. Measurement of SOD Activity

The method of determining SOD activity has been previously described [34]. Briefly, superoxide radicals are generated by the xanthine and xanthine oxidase reaction. The amount of superoxide radical produced is measured by **2**-(**4**-iodophenyl)-**3**-(**4**-nitrophenol)-**5**-phenyl tetrasodium chloride [35], which reacts with a superoxide radical to form a red formazan dye. SOD activity is then determined by the grade of the reaction’s inhibition by measuring its absorbance at 550 nm. The standard calibration curve of inhibition percentage by standard solutions and concentrations (U/mL) was used to evaluate total SOD activity.

### 2.6. Measurement of GSH-Px Activity

GSH-Px activity was measured indirectly by measuring the rate of oxidized glutation (GSSG) formation [36]. The principle of the method was as follows: GSH-Px catalyzes the oxidation of GSH with synthetic cumen hydroperoxide to GSSG. In the presence of glutathione reductase and NADPH, GSSG is immediately converted to the reduced form with a concomitant oxidation of NADPH to NADP^+^. The rate of NADPH oxidation was measured at absorbance 412 nm and is proportional to the activity of GSH-Px.

### 2.7. Statistical Analysis

All spreadsheet calculations were performed using Microsoft Excel™ 2007, Sigma Plot 12.0 (Systat Software Inc., San Jose, CA), Origin Pro 8.5.1(Origin Lab Corporation, Northampton, MA, USA) or the software Prism 6.0 (GraphPad Software Inc., San Diego, CA, USA). All datasets of different treatments were tested for statistically significant differences using one-way analysis of variance. Dunnett’s test was used to identify significant differences between treatments and controls.

## 3. Results

### 3.1. Cytotoxic Effects of Soluble Sulfide

**Figure 1:**
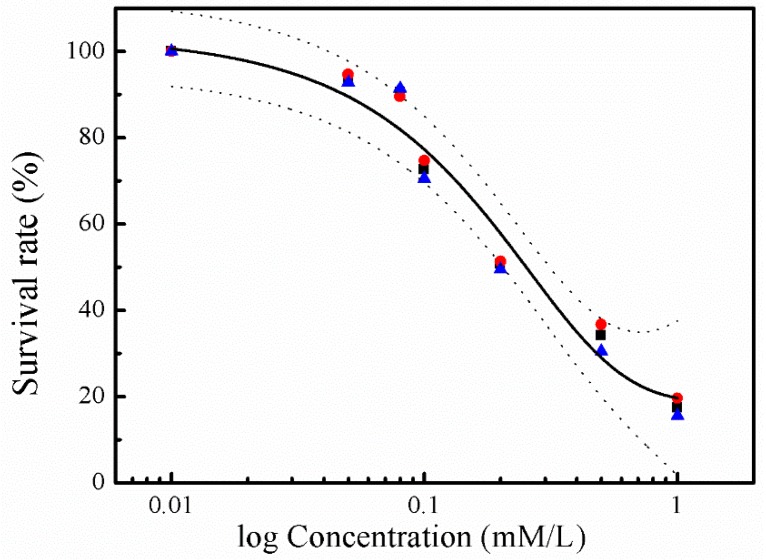
Concentration–response curves for the cytotoxicity of sulphide solution. The experimental data depicts viability of hepatocyte LO2 cells exposed to sulphide solutions after 24 h of incubation (1st replicate: red roundness; 2nd replicate: blue triangle; and 3rd: black square). The regression curves (black lines) are shown with their 95% confidence intervals (dashed lines), in which the top and bottom of the curve was set to 0% and 100%, respectively.

Cytotoxicity of sulfide was investigated in human hepatocyte LO2 cells using the MTT assay. Incubation of LO2 cells with sulfide solution at concentrations ranging from 0.01 to 1.0 mM/L resulted in a marked and concentration-dependent reduction in their viability (Figure 1). No cytotoxicity effect was found at 0.01 mM/L, while the strongest cytotoxicity effect was recorded at 1.0 mM/L, with the average survival rate accounting for 17.5%.

### 3.2. Oxidative Stress Effects of Soluble Sulfide

#### 3.2.1. Hydroxyl Radical Production by Soluble Sulfide

**Figure 2:**
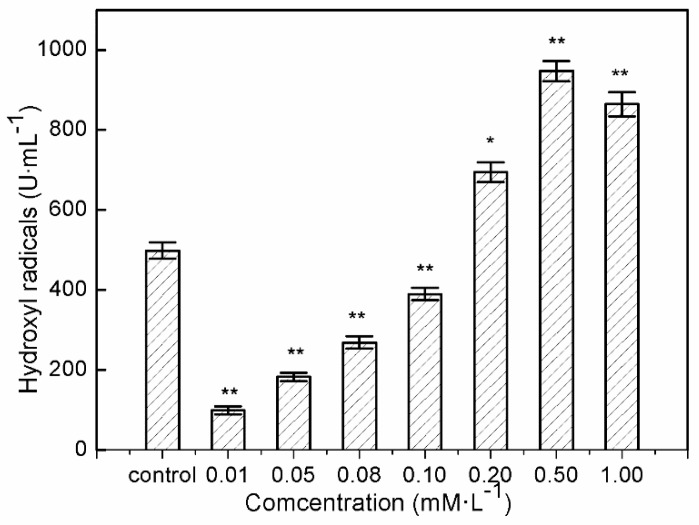
Hydroxyl radical generation by human hepatocytes LO2 after 24 h of exposure to sulfide solution. The values were determined by the Fenton reaction using a deoxyribose method. Data are given as means of three replicates ± SD. * *p* < 0.05, ** *p* < 0.01: significant differences from controls.

Significant decrease in intracellular hydroxyl radical formation was found in human hepatocytes LO2 after 24 h of exposure at 0.01–0.1 mM/L sulfide solutions (Figure 2). The significant decline in hydroxyl radicals in the treated hepatocytes after exposure to 0.01 mM/L sulfide solution was nearly one fifth that of the control. This provided evidence of hydroxyl radical inhibition in LO2 cells after exposure to soluble sulfide, and the protective effects of soluble sulfide against oxidative stress. While LO2 exhibited virtually equal hydroxyl radical levels when exposed to a 0.1 mM/L sulfide solution, and even significantly higher hydroxyl radical levels after treatment with higher concentrations of sulfide solution, i.e., 0.1–1.0 mM/L. 

#### 3.2.2. SOD and GSH-Px Activities Induction by Soluble Sulfide

**Figure 3:**
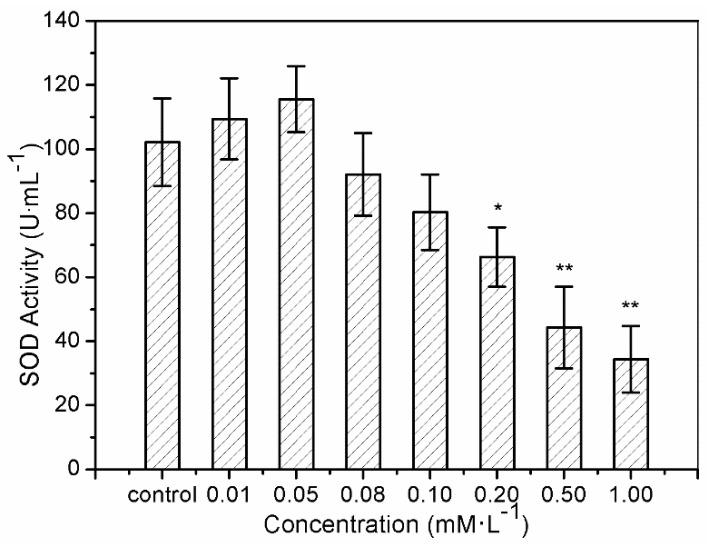
Superoxide dismutase (SOD) activity of the human hepatocytes LO2 after 24 h of exposure to sulfide solutions. The values were determined by the xanthine and xanthine oxidase reaction using 2-(4-iodophenyl)-3-(4-nitrophenol)-5-phenyl tetrasodium chloride [34]. Data are given as means of three replicates ± SD. * *p* < 0.05, ** *p* < 0.01: significant differences from controls.

**Figure 4:**
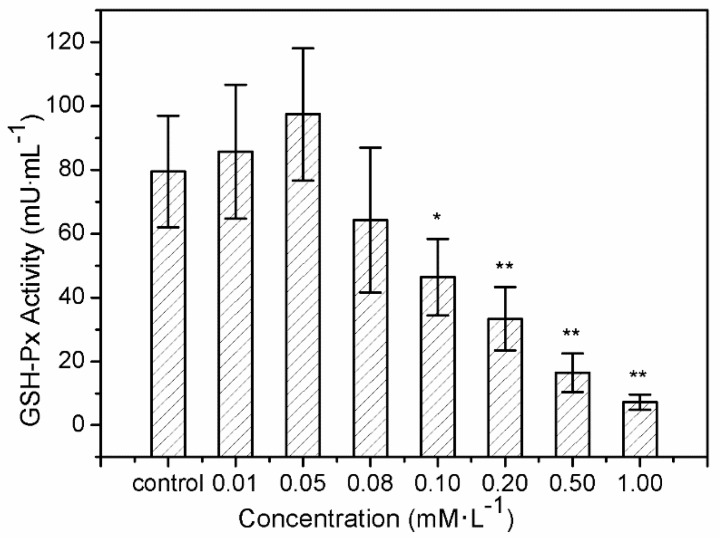
Glutathione peroxidase (GSH-Px) activity of the human hepatocytes LO2 after 24 h of exposure to sulfide solutions. The values were determined by measuring the rate of formation of oxidized glutation (GSSG). Data are given as means of three replicates ± SD. * *p* < 0.05, ** *p* < 0.01: significant differences from controls.

The results of SOD activity and GSH-Px activity showed similar trends. After treatment with sulfide solution at concentrations of 0.01 and 0.05 mM/L for 24 h, SOD activity in LO2 cells increased slightly from 109.43 ± 12.6 U/mL to 115.54 ± 10.3 U/mL, compared to the control (102.14 ± 13.7 U/mL) (Figure 3). SOD activity then declined continuously from 92.06 ± 12.9 U/mL to 34.42 ± 10.4 U/mL, under exposures to 0.08–1.0 mM/L. GSH-Px activity increased from 85.72 ± 21.02 U/mL to 97.47 ± 20.72 U/mL after 0.01 and 0.05 mM/L sulfide exposure for 24 h, and that for the treatments of 0.08–1.0 mM/L declined significantly from 64.26 ± 22.76 U/mL to 7.22 ± 2.35 U/mL (Figure 4).

## 4. Discussion

Soluble sulfide reduces cell viability to a considerable extent only at concentrations above 0.1 mM/L. Co-incubation with sulfide at 1.0 mM/L induced almost 90% viability loss for hepatocytes in the current study (Figure 1), which is in agreement with an erythrocytes study, where almost no cell remained viable after sulfide exposure at 1.5 mM/L [37]. Even though the specific toxic mechanism of sulfide in LO2 cells is still unclear, studies have revealed that the molecular mechanisms underlying the toxicological effects of H_2_S are mostly attributed to mitochondrial poisoning [38,39]. Treating H_2_S poisoning may benefit from interventions minimizing ROS-induced damage and reducing mitochondrial damage [40,41]. A NaHS study has shown that sulfide solutions have complex effects on the electrophysiological properties of neuronal membranes, and an array of K^+^ conductance at toxicologically relevant concentrations [42]. Furthermore, the concentration–response curve of LO2 cells viability in the current study (Figure 1) indicates a gradual appearance of toxicity with the increase in sulfide concentration. These cytotoxic effects are consistent with an erythrocytes study, in which the fraction of viable cells was decreased with the increase of sulfide concentration ranging from 0.18 to 4.8 mM/L [37]. These results indicate that normal human hepatocyte LO2 cells show similar toxicity to erythrocytes. Thus, the environment and health risk assessment and management should pay more attention to the toxic effects when investigating soluble sulfide exposure in different tissues or cells, especially for the occupational exposure for individuals such as miners [43].

As described in a study by Turrens [44], high levels of ROS induced detrimental effects by damaging cell structures, lipids, DNA, and proteins which ultimately lead to cell death, but low “physiological” levels of ROS play important roles in signal transduction and are involved in the communication between nucleus and mitochondria [45,46]. ROS are reduced derivatives of molecular oxygen (e.g., O^2−^·, H_2_O_2_, ·OH, ferryl, peroxyl, and alkoxyl), being produced during energy metabolism or the defense process against infection in cells and tissues [47]. Of all generated ROS in biological systems, the hydroxyl radical is the most reactive oxygen radical. When the hydroxyl radical is generated in excess, or the cellular antioxidant defense is deficient, oxidative stress and oxidative damage to lipid, DNA, protein, and other key molecules are caused via stimulating free radical chain reactions with proteins, lipids, and nucleic acids [48]. Evidence of hydroxyl radical decline at sulfide solution concentrations of 0.01–0.1 mM/L on LO2 cells suggested possible mechanisms of anti-oxidative stress which were induced by sulfide solutions in the present study, which could help during the process of drug development to treat or relieve oxidative damage-induced disease.

The superoxide anion (O^2−^), as the first free radical of ROS generated in vivo, can initiate a variety of oxidative damage responses to phospholipids, proteins, and nucleic acids by generating many kinds of oxygen free radicals, causing degenerative diseases and aging [35,49]. SOD, an O^2−^ level downregulation catalyzing enzyme, is necessary for almost all organisms living in the presence of oxygen [26]. Therefore, SOD activity is often used to detect O^2−^ defense in cells based on the superoxide reacts. The phenomena of increase of SOD activity after 24 h of exposure to sulfide solutions at 0.01 and 0.05 mM/L in the current study indicates the protective function of soluble sulfide against oxidative stress, which gives further evidence of the medicinal value of low dose soluble sulfide. These results are consistent with a cardiomyocytes study, in which hydrogen sulfide decreased the levels of ROS by inhibiting mitochondrial complex IV and increasing SOD activities under ischemia/reperfusion [50]. While the study from H_2_S metabolism reported that H_2_S (or H_2_S donors) may interact/react with SOD cysteines to affect catalytic activity or directly contribute to sulfide metabolism [51], which differs from the well-known SOD-mediated dismutation of two O^2−^ to form H_2_O_2_ and O_2_. 

H_2_O_2_, as the second free radical of ROS generated in vivo, must be eliminated by the GSH-Px in the current study. GSH-Px can change lipid hydroperoxides and free hydrogen peroxide into their corresponding alcohols and water, thus protecting the organism from oxidative damage [52]. GSH-Px is hence widely used as another enzyme for detecting oxidative stress defense. The increase in GSH-Px activity provided further evidence of soluble sulfide function against oxidative stress. Moreover, recent studies provide experimental evidence of the role of antioxidant enzymes such as GSTP1 in many tissues, functioning as regulator of pro/anti apoptotic pathways [53,54,55]. Hydrogen sulfide regulates oxidative stress by GSH-Px through Sirtuin-1 pathway to protect against apoptosis, which was also shown in the context of cardiomyocytes [56]. The decline of GSH-Px activity at sulfide concentrations of 0.08–1.0 mM/L could be explain that the increase in ROS may be the reason for the inhibition of GSH-Px activity [57]. 

The present study shows that sulfide solutions at concentrations of 0.08–1.0 mM/L decreased SOD and GSH-Px activities after 24 h exposure, and simultaneously decreased LO2 cell viability (Figure 1, Figure 3 and Figure 4). This decline in SOD, GSH-Px, and cell survival rate suggests that oxidative damage could be a possible mechanism of cytotoxicity induced by soluble sulfide in LO2 cells. A necrosis study also reported that disturbed SOD could induce reverse cytoprotective properties, destabilizing the mitochondrial membrane system and promoting cell death [32]. Hydroxyl radical levels also increased at these concentration ranges in the current study (Figure 2), which gives further evidence of the oxidative damage at high concentrations of soluble sulfide on LO2 cells. Previous studies also provide experimental evidence that oxidative stress-mediated apoptosis could be activated by caspase-3, and an imbalance between Bax and Bcl-2 expression with different level bioassays [58]. Moreover, NaHS has been shown to have anti-apoptotic and anti-inflammatory signaling potential via mechanisms involving Nrf-2 by direct and indirect anti-oxidant activities [59]. While on the contrary, sulfide solutions at concentrations of 0.01 and 0.05 mM/L induced the increase of SOD and GSH-Px activities, and decrease in the hydroxyl radical level on LO2 cells (Figure 2, Figure 3 and Figure 4). Recent studies reported that H_2_S can protect neurons from oxidative stress by restoring glutathione levels for the hypochlorous acid-mediated oxidative damage in the brain [21],t renal cell carcinoma, transitional cell carcinoma in the kidneys, and urinary bladder tumors [54]. Chemicals at low concentrations exhibit protective effects by upregulating cellular antioxidant enzymes such as SOD, GSH-Px, and CAT, which has been confirmed recently with qPCR assays [60], western blot analysis, and immunofluorescence assays [61]. A biphasic response of low concentration stimulation, high concentration inhibition for SOD activity and GSH-Px activity and low concentration inhibition, high concentration stimulation for hydroxyl radical levels exhibited a clear hormesis-effect after exposure to soluble sulfide on LO2 cells (Figure 2, Figure 3 and Figure 4) [62], which is consistent with a luminescent bacteria toxicity study, in which an opposite hormesis-effect appeared at the low concentrations of sulfide exposure [3]. These clear hormesis-effects provide further evidence of the protective ability of low-dose soluble sulfide and the toxicity of high-dose soluble sulfide, which should be considered by pharmaceutical researchers and public health research.

## 5. Conclusions

Soluble sulfide solutions at concentrations of 0.01–1.0 mM/L showed concentration-dependent cytotoxicity in the hepatocyte line LO2. While clear hormesis-effects were evident during the oxidative stress effect evaluation by the hydroxyl radical, the superoxide dismutase (SOD), and the glutathione peroxidase (GSH-Px) assays. The results indicate the protection of soluble sulfide at 0.01–0.1 mM/L against oxidative stress. Furthermore, the decrease in SOD and GSH-Px, in addition to the increase in hydroxyl radical by soluble sulphide suggests that oxidative damage could be a possible mechanism of cytotoxicity induced by sulphide in LO2 cells. However, further studies must analyze the different biological-level effects under oxidative stress conditions must be analyzed to demonstrate the protective role of soluble sulfide. The current study can serve as a step towards further demonstrating the mechanisms of soluble sulfide toxicity. These efforts provide the scientific underpinnings and regulatory reference for sulfide contaminations in the context of public and environmental health.
